# A trait‐based approach reveals the feeding selectivity of a small endangered Mediterranean fish

**DOI:** 10.1002/ece3.2117

**Published:** 2016-04-12

**Authors:** Pablo Rodríguez‐Lozano, Iraima Verkaik, Alberto Maceda‐Veiga, Mario Monroy, Adolf de Sostoa, Maria Rieradevall, Narcís Prat

**Affiliations:** ^1^ Freshwater Ecology and Management (F.E.M.) Research GroupDepartament d'EcologiaFacultat de BiologiaUniversitat de BarcelonaAvda. Diagonal, 643E‐08028BarcelonaSpain; ^2^ Departament of Integrative EcologyEstación Biológica de DoñanaAvda. Américo Vespucio, s/nE‐41092SevillaSpain; ^3^ Institut de Recerca de Biodiversitat (IRBio)Universitat de BarcelonaE‐08028BarcelonaSpain; ^4^ Departamento de Biología AmbientalUniversidad Jorge Tadeo LozanoBogotáColombia; ^5^ Departament de Biologia Animal (Vertebrats)Facultat de BiologiaUniversitat de BarcelonaAvda. Diagonal, 643E‐08028BarcelonaSpain

**Keywords:** Fish diet, food webs, functional traits, intermittent streams, macroinvertebrates, species extinction

## Abstract

Functional traits are growing in popularity in modern ecology, but feeding studies remain primarily rooted in a taxonomic‐based perspective. However, consumers do not have any reason to select their prey using a taxonomic criterion, and prey assemblages are variable in space and time, which makes taxon‐based studies assemblage‐specific. To illustrate the benefits of the trait‐based approach to assessing food choice, we studied the feeding ecology of the endangered freshwater fish *Barbus meridionalis*. We hypothesized that *B. meridionalis* is a selective predator which food choice depends on several prey morphological and behavioral traits, and thus, its top‐down pressure may lead to changes in the functional composition of in‐stream macroinvertebrate communities. Feeding selectivity was inferred by comparing taxonomic and functional composition (13 traits) between ingested and free‐living potential prey using the Jacob's electivity index. Our results showed that the fish diet was influenced by 10 of the 13 traits tested. *Barbus meridionalis* preferred prey with a potential size of 5–10 mm, with a medium–high drift tendency, and that drift during daylight. Potential prey with no body flexibility, conical shape, concealment traits (presence of nets and/or cases, or patterned coloration), and high aggregation tendency had a low predation risk. Similarly, surface swimmers and interstitial taxa were low vulnerable to predation. Feeding selectivity altered the functional composition of the macroinvertebrate communities. Fish absence favored taxa with weak aggregation tendency, weak flexibility, and a relatively large size (10–20 mm of potential size). Besides, predatory invertebrates may increase in fish absence. In conclusion, our study shows that the incorporation of the trait‐based approach in diet studies is a promising avenue to improve our mechanistic understanding of predator–prey interactions and to help predict the ecological outcomes of predator invasions and extinctions.

## Introduction

Studies on fish feeding ecology are crucial to understanding trophic interactions and energy fluxes in ecosystems and help develop better conservation and management strategies for species and ecosystems (Braga et al. 2012). Current biodiversity loss is biased toward species in the higher trophic levels (Duffy 2002; Schneider and Brose 2013), freshwater fish being among the most threatened fauna worldwide, particularly the small‐bodied ones (Jenkins 2003; Olden et al. 2007). Small‐bodied fish species often act as top predators in many freshwater systems (i.e., intermittent rivers, headwater permanent streams, and ponds) (Meyer et al. 2007; Reich et al. 2010; Brucet et al. 2012) and, consequently, their extirpation may trigger multitrophic impacts in food webs and major changes in ecosystem functions (Rodríguez‐Lozano et al. 2015). Our understanding of how these species influence food webs remains limited, mainly because most of the feeding ecology studies in freshwater fish have been focused on commercial and recreational species (Braga et al. 2012).

Traditionally, studies on selective predation have used a taxon‐based approach, even though consumers do not have any reason to select their prey using a taxonomic criterion. Moreover, prey assemblages vary over space and time, which makes taxon‐based studies assemblage‐specific. According to the optimal foraging theory (OFT), predators select their prey in order to maximize their net rate of energy gain in relation to the energetic costs (Pyke 1984). In this trade‐off, prey size has been identified as the primary determinant of predator choice, as it reflects well the costs (e.g., handling time) and benefits of foraging (e.g., prey energy content) (Werner and Hall 1974; Woodward and Warren 2007). However, other traits, either morphological (e.g., concealment and body shape) or behavioral (e.g., drift tendency and prey movements), may also determine the predator optimal foraging strategy (Rader 1997; de Crespin de Billy and Usseglio‐Polatera 2002; Allan and Castillo 2007; Klecka and Boukal 2013). In this line, a trait‐based approach has been further applied in a descriptive way to study fish diet (e.g., de Crespin de Billy and Usseglio‐Polatera 2002; Sánchez‐Hernández et al. 2012) and only a few recent studies have examined the selectivity of predatory fish on several prey traits (Green and Côté 2014; Worischka et al. 2015). This new framework raises as a promising avenue to better understand predator–prey interactions and may also help predict the consequences of species extirpations and invasions on food webs.

The present experimental study aims of illustrating the benefits of the trait‐based approach to assessing food choice and of testing the hypothesis that alterations in the density of a small‐bodied fish can affect the functional structure of macroinvertebrate communities. We used *Barbus meridionalis* (A. Risso, 1827) as a case study for three main reasons. First, it can be the major top‐down control of the aquatic community in intermittent streams (Rodríguez‐Lozano et al. 2015). Second, there is an urgent need to understand its role in ecosystems, as it is listed as “vulnerable” in the Spanish Red Book (Doadrio 2001), “near threatened” by the IUCN, and is also included in the EU's Habitats Directive and in the Bern Convention, due to many anthropogenic impacts, including water abstraction, dam construction, biological invasions, and alterations in habitat (Doadrio et al. 2011; Maceda‐Veiga 2013). Finally, the scarce information about its feeding ecology (García‐Berthou 1994; Mas‐Martí et al. 2010; Doadrio et al. 2011) allowed us to predict that their potential prey (e.g., midges, mayflies) vary across its distribution range from NE Spain to SE France. Such information was also important to frame our specific hypothesis: (1) *B. meridionalis* is a selective predator (the proportion of taxa consumed differs from taxa density in the system), (2) its selectivity increases at low densities, (3) its selectivity depends on several prey morphological and behavioral traits (all rationale listed in Table 1), and thus, (4) fish presence and density matters for the functional structure of stream macroinvertebrate communities.

**Table 1:** *A priori* hypotheses of expected influence of morphological and behavioral traits of macroinvertebrates on macroinvertebrate vulnerability to predation by *Barbus meridionalis*. Categories and codes used in analyses and graphics are also indicated

Trait	Rationale	Categories	Codes
Potential size (mm)	The predator may prefer a particular size range because prey size is a trade‐off among costs and benefits of foraging	≤2.5	<2.5
		2.5–5	2.5–5
		5–10	5–10
		10–20	10–20
		20–40	20–40
		40–80	40–80
		>80	>80
Body shape (including cases/tubes)	Body shape can affect handling efficiency	Cylindrical	cyl
		Spherical	sph
		Conical	con
		Flattened	flat
Body flexibility (including cases/tubes)	Higher body flexibility may be preferred because it compromises lower handling time	None	f.no
		Weak	f.weak
		High	f.high
Concealment	Prey concealment (colored patterns, accessories) may reduce vulnerability to predation	Fixed accessory (nets, retreats)	net
		Movable accessory (cases, tubes)	case
		Solidly colored	c.sol
		Variable	c.var
		Patterned	c.patt
Morphological defenses	Morphological defenses may reduce vulnerability to predation	Cerci, silk, spine	def.sp
		None	def.no
Locomotion and substrate relation	Crawlers, burrowers and attached organisms may be more vulnerable to predation because the fish foraging preferences (benthic)	Surface swimmer	surf.s
		Full water swimmer	wat.s
		Crawler	craw
		Burrower	burw
		Interstitial	int
		Attached	att
Tendency to drift in the water column	Drift tendency may increase vulnerability to predation	None	d.no
		Weak	d.weak
		Medium	d.med
		High	d.high
Diel drift behavior	Nocturnal drift may reduce vulnerability to predation because foraging efficiency can decrease with a decrease in luminosity	None	none
		Nocturnal	noct
		Dawn	dawn
		Daylight	d.light
		Twilight	t.light
Agility	High agility may increase encounter rate with the predator, increasing prey vulnerability to predation	None (sluggish)	a.no
		Weak	a.weak
		High	a.high
Movement frequency	Continuous movement may increase vulnerability to predation because it may reduce hunting time	Continuous	cont
		Discontinuous	disc
Trajectory on the bottom substratum or in the drift	Random trajectory may reduce vulnerability to predation because it may increase hunting time	None	t.no
		Linear	t.lin
		By random	t.rand
		Oscillatory	t.oscil
Aggregation tendency	Aggregation may increase vulnerability trough increasing conspicuous to predators, but may be also an antipredator strategy	Weak	ag.weak
		High	ag.high
Feeding habits	Habitat use by the predatory fish may affect differently to the potential prey depending on prey feeding habits	Absorber	abs
		Deposit feeder	dpfd
		Shredder	shrd
		Scraper	scrp
		Filter‐feeder	filt
		Piercer	pier
		Predator	pred
		Parasite	par

## Materials and Methods

### Study area

The study was carried out in the Vall d'Horta stream within the protected area of Sant Llorenç del Munt i l'Obac Natural Park (50 km inland from Barcelona City, NE Spain). This area has calcareous geology and is under the Mediterranean‐climate domain, with mild winters and warm springs and summers. This area is dominated by Holm Oak (*Quercus ilex* L.) and Aleppo pine (*Pinus halepensis* Miller) forests and Mediterranean shrubs (see Bonada et al. 2007 for a detailed site description). In intermittent streams, *Barbus meridionalis* can be the major top‐down control of the aquatic community (Rodríguez‐Lozano et al. 2015). In August 2003, a wildfire burned a forested area of 4543 ha and, consequently, *B. meridionalis* was locally extinct in some streams, most likely due to water quality deterioration (Vila‐Escalé et al. 2007). The fish population has not recovered since then, possibly due to the presence of natural and human barriers downstream.

### Field and laboratory work

In this study, we used the benthic macroinvertebrates and gut content data from a previous study, which also provides full details on the experimental design (Rodríguez‐Lozano et al. 2015). We carried out an enclosure experiment in the Vall d'Horta stream (41°40′24″N, 2°02′4″E; Altitude: 480 m.a.s.l.), a first‐order stream in the Besòs river basin. The experiment was run for 5 weeks in late spring in 2010 before pool disconnection (discharge averaged 15.7 ± 0.9 l s^1^; water velocity <1 cm s^−1^). The mesocosm experiment consisted of large cages (100 × 100 cm surface, 70 cm height) of 10‐mm mesh size, enabling macroinvertebrates to pass through. We randomly assigned three fish densities to cages (3 cages/treatment) in order to simulate: no fish, fish at low density (known prefire fish density; 2 individuals m^−2^, Adolf de Sostoa *pers. comm*.), and fish at high density (twofold increase in prefire density; 4 individuals m^−2^). Each cage contained four trays (with stones and glass tiles) that were left for 3 weeks in order to allow for the establishment of the macroinvertebrate community. Throughout the mesocosm experiment, we found 81 taxa (76 aquatic invertebrates, 1 amphibian, and 4 terrestrial invertebrates). Macroinvertebrate communities in the mesocosms were similar to those found during previous research in the stream (Verkaik et al. 2013) (for further description of the mesocosm experiment, see Rodríguez‐Lozano et al. 2015).

Fish were caught using electrofishing downstream from our study site. Eighteen individuals (6 for the low‐density treatment and 12 for the high‐density treatment) were size‐matched (total length: 101.8 ± 2.6 mm; weight: 2.3 ± 0.2 g mean ± SE) and kept for observation, caged in the stream, for 24 h prior to the experiment. After 2 weeks in the mesocosms, fish individuals were euthanized using an overdose of the anesthetic MS‐222^®^ (Tricaine methane‐sulfonate, Sigma‐Aldrich, St. Louis, MO), measured (total length, ±1 mm), weighed (±0.01 g), dissected, and the entire guts (full gastrointestinal tracts) were preserved in 4% formalin. To quantify the potential prey, the content of each tray was carefully sieved through a 250‐*μ*m mesh and individually preserved in 4% formalin.

In the laboratory, we sorted and counted all macroinvertebrates in gut and benthos samples under the stereomicroscope. All taxa were identified to the genus level, with the exception of some dipterans (family level) and Oligochaeta, Ostracoda, Cladocera, Copepoda, Hydracarina, and terrestrial invertebrates. The same taxonomic resolution was used for free‐living and ingested prey.

### Data analysis

**Figure 1:**
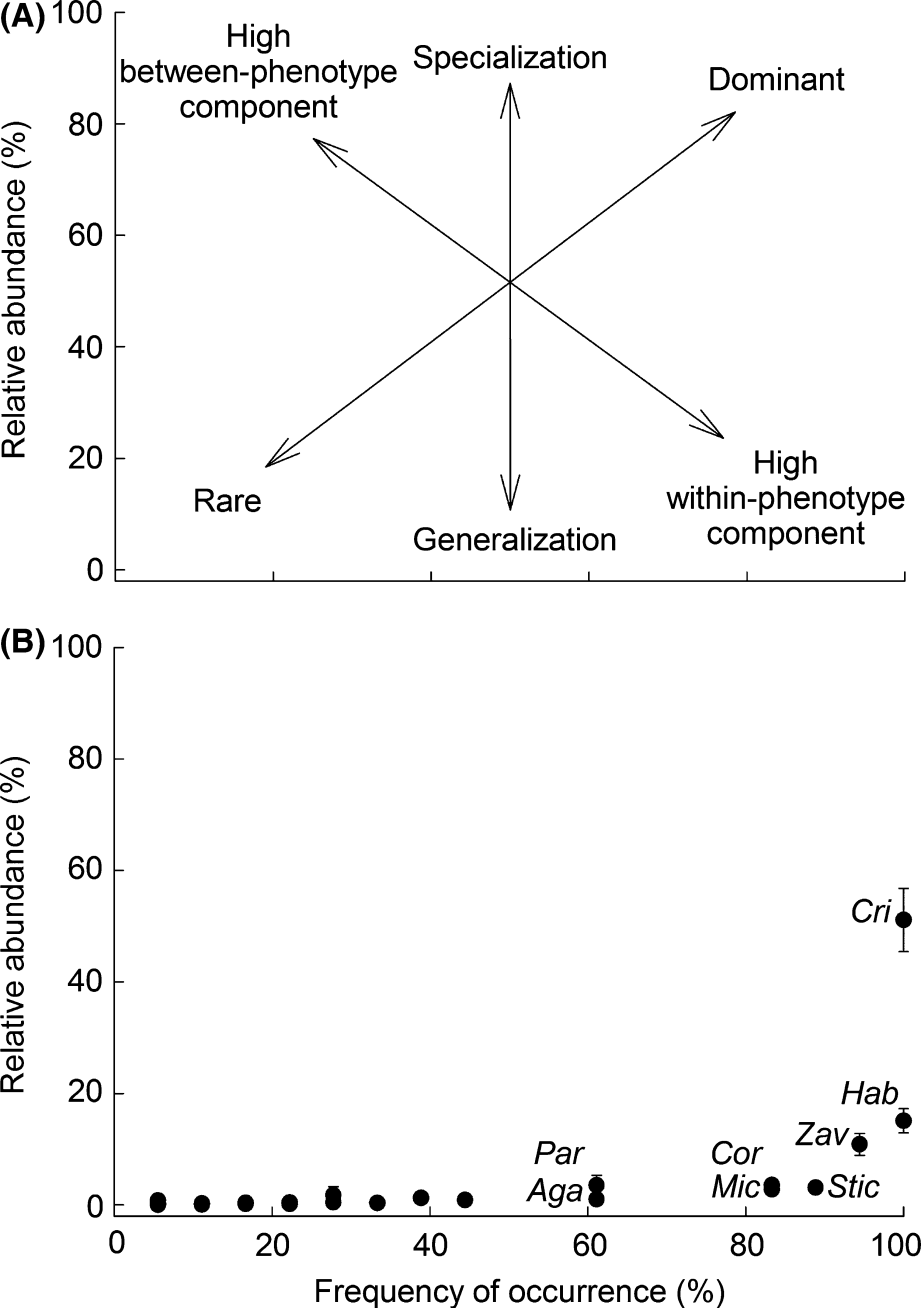
Graphical evaluation of the feeding strategy according to Amundsen et al. (1996). (A) Explanatory diagram about feeding strategy (generalization vs. specialization), prey importance (dominant vs. rare), and phenotypic contribution of niche width (within‐phenotype vs. between‐phenotype). (B) Relationship between relative abundance (%) and frequency of occurrence (%) of food categories in the *Barbus meridionalis* gut contents. The written food categories represent the most important prey items: Cri, *Cricotopus* spp.; Hab, *Habrophebia* sp.; Zav, *Zavrelimyia* sp.; Stic, *Stictonectes* sp.; Cor, *Corynoneura* spp.; Mic, *Microtendipes* sp.; Par, *Parasigara* sp.; Aga, *Agabus* sp.

We used a taxonomic and functional approach to assess the diet of *B. meridionalis*. For the taxonomic approach, the relative importance of each taxon was estimated by determining the relative abundance of each prey item (i.e., number of individuals of a prey in a gut divided by the total number of individuals) and their frequency of occurrence (the percentage of guts in which a prey was present), both expressed as a percentage. The graphical method of Amundsen et al. (1996) was used to visualize the prey importance, the feeding strategy, and the phenotypic contribution to the niche width (Fig. 1A). Diet diversity was calculated using the Shannon–Wiener index (*H*′ = −∑*P*_*i*_log_10_ *P*_*i*_, where *P*_*i*_ is the proportion of the diet that is represented by prey item *i*), and the specialization in the diet was evaluated using Pielou's evenness index (*J* = *H*′/*H*′_max_), considering that values close to zero indicate a stenophagous diet and those closer to one indicate a euryphagous diet (Oscoz et al. 2005).

To analyze the taxonomical feeding selectivity of *B. meridionalis*, we compared macroinvertebrates found in the gut contents with the macroinvertebrate community. Feeding selectivity was measured using Jacob's index of electivity *D* (Jacobs 1974), calculated as *D* = *r*−*p*/(*r* + *p*−2*rp*), where *r* is the proportion of the diet accounted for by a given prey taxon, and *p* is the proportion of the taxon per predator cage accounted for by that taxon. *D* varies from −1 to 0 for negative selection and from 0 to +1 for positive selection. To test whether selectivity significantly deviated from 0, a one‐sample nonparametric test (Wilcoxon Signed Rank test) was used, as data were not normally distributed. As chironomids are rarely identified to the genus level, we explored the importance of taxonomic resolution on the measure of feeding selectivity by comparing outputs for chironomids at the family, subfamily, and genus level. This family was also selected because from our experience, Chironomidae is the most diverse family in these streams.

The importance of fish density in diet diversity and feeding selectivity was analyzed using the nonparametric Mann–Whitney test. Besides, we used permutational multivariate analysis of variance (PERMANOVA, “Adonis” function in R) on the Bray–Curtis distance matrix, after the log‐transformation of the macroinvertebrate abundance data, to test differences in macroinvertebrate composition of gut contents between treatments.

For the trait‐based approach, we used 13 macroinvertebrate traits with 55 trait categories (see Table 1 and Table S1) from public depositories (de Crespin de Billy 2001; Tachet et al. 2010). A score was assigned to each taxon for each trait using a fuzzy coding approach (e.g., a score between 0 and 5, with “0” indicating “no affinity” to “5” indicating “high affinity”) (Chevenet et al. 1994). These affinity scores were rescaled into relative proportions within each trait (between 0 and 1). As some genera (mostly Chironomidae) were not included in public trait databases, these were coded using the following: other published information (e.g., Puntí et al. 2009), the available information at subfamily or family level, the mean of other genera values within the same family, and the personal experience of the senior authors of this study. Ostracoda, Cladocera, Copepoda, Hidracarina, tadpoles, and terrestrial invertebrates were not included in the trait analysis due to the lack of trait information.

We compared the traits of the macroinvertebrates found in the gut contents with the traits of the macroinvertebrate communities in the mesocosms. To this aim, we created a “diet samples x traits” array and a “mesocosm samples x traits” array (only with the samples from mesocosms with fish individuals). For the arrays, the fuzzy‐coded categories of each of the 13 variables were weighted with the relative abundances of the taxa in the respective samples. To compare the mesocosm with the diet samples, we combined the two arrays to one joint dataset and we performed a fuzzy principal component analysis (FPCA). After that, we inferred the selection of *B. meridionalis* for prey traits, as we did for the taxonomical feeding selectivity. For each trait category, Jacob's electivity index was calculated and Wilcoxon signed rank test was computed to test whether selectivity was statistically significant.

In order to test whether the presence and density of *Barbus meridionalis* affected the trait composition of the macroinvertebrate community, we performed a FPCA on the “mesocosm samples x traits” array, including the data of the mesocosms lacking fish individuals. We only included the information regarding the 10 traits that were significantly selected by *B. meridionalis*, following Worischka et al. (2015). All statistical analyses were performed in R 2.15.2. (R Core Team 2012).

### Ethical note

This study was approved by the Autonomous Government of Catalonia (Generalitat de Catalunya) and the Natural Parks Department of the Government of Barcelona (Diputació de Barcelona). This authorization only enabled us to sacrifice 18 fish that were euthanized following the procedure used in the aquatic animal facility at the University of Barcelona. All efforts were made to minimize animal stress, and individuals captured represented 10% of the fish in the donor population.

## Results

### Taxon‐based diet analysis

Overall, 38 different taxa were found in *B. meridionalis* guts. Mean prey abundance in gut contents was 161 ± 30 (mean ± SE) individuals. Chironomids and ephemeropterans were the most frequent groups in fish gut contents, but other taxonomical groups were also frequently found (i.e., coleopteran larvae, heteropterans, gastropods, and odonates) (Fig. 1 and Table S2). *Barbus meridionalis* presented a generalized diet, but three prey taxa (*Cricotopus* spp., *Habrophlebia* sp., and *Zavrelimyia* sp.) were the most important (above the diagonal from the upper left to the lower right; Fig. 1). Fish individuals had a high within‐phenotype component (Fig. 1), that is, most individuals utilized many resources types simultaneously. Diet diversity (0.62 ± 0.04) and the evenness index (0.68 ± 0.05) further supported that these fish tended to be euryphagous. Moreover, fine sediment, that is, detritus and sand, was barely present in all gut contents, but we did not quantify it.

**Figure 2:**
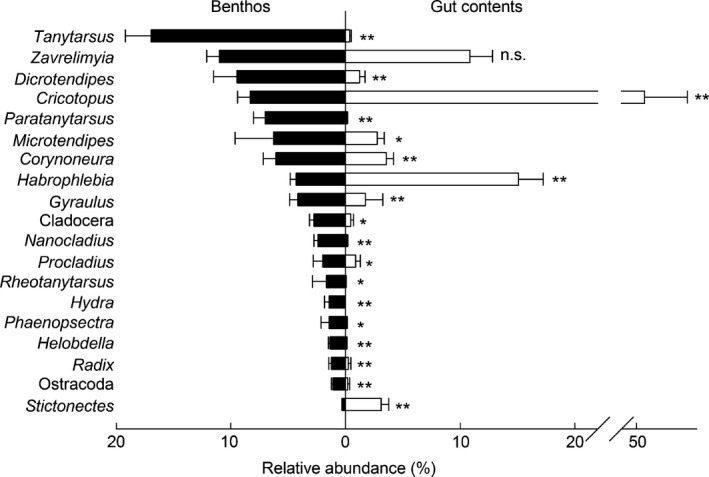
Relative abundance of the mesocosm taxa (left) compared to its relative abundance in gut contents (right) taxa. The taxa are ordered by abundance found in the mesocosm samples, and only the most abundant ones (>1%) are presented. The statistically significant thresholds are as follows: *P < 0.05, **P < 0.01.

**Figure 3:**
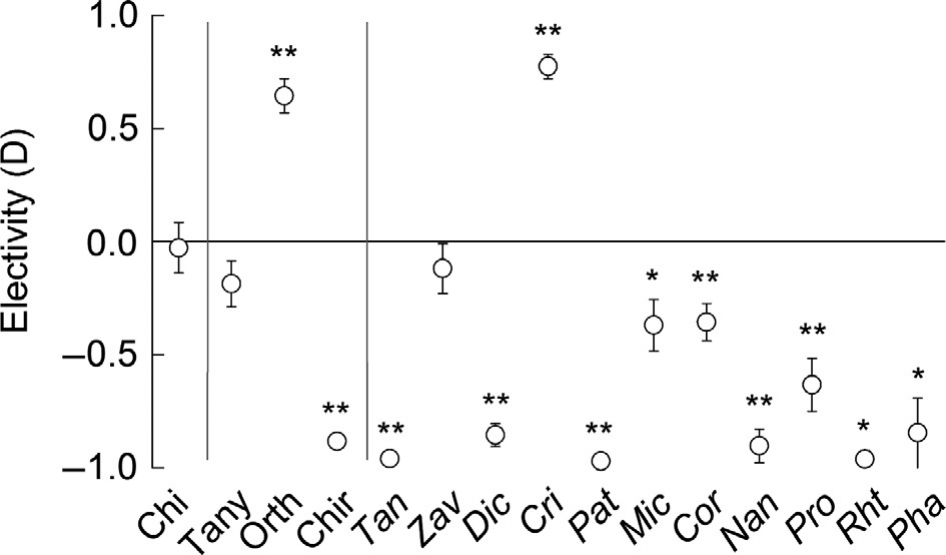
Selectivity of *Barbus meridionalis* for the Chironomidae family, subfamilies, and genera. Chi, Chironomidae family; Tany, Tanypodinae subfamily; Orth, Orthocladinae subfamily; Chir, Chironominae subfamily; Tan, *Tanytarsus* sp.; Zav, *Zavrelimyia* sp.; Dic, *Dicrotendipes* sp.; Cri, *Cricotopus* spp.; Pat, *Paratanytarsus* sp.; Mic, *Microtendipes* sp.; Cor, Corynoneura spp.; Nan, *Nanocladius* sp.; Pro, *Procladius* sp.; Rht, *Rheotanytarsus* sp.; Pha, *Phaenopseptra* sp. The different genera are ordered by their abundance found in the mesocosms. The statistically significant thresholds are: *P < 0.05, **P < 0.01.

The prey electivity index revealed that *B. meridionalis* did not feed on many potential prey (*D* = −0.70 ± 0.02; *P* < 0.001). Surprisingly, these discarded taxa included some of the most abundant chironomids, such as *Tanytarsus* sp. and *Dicrotendipes* sp. (Figs 2 and 3). In contrast, other abundant taxa, such as *Cricotopus* spp. (*D* = 0.77 ± 0.05; *P* < 0.001), *Habrophlebia* sp. (*D* = 0.44 ± 0.10; *P* < 0.001), and *Stictonectes* sp. (*D* = 0.57 ± 0.14; *P* < 0.03), were highly positively selected by *B. meridionalis* (Fig. 2). In general, *B. meridionalis* ate less chironomids than were in the benthos, with the exception of *Cricotopus* spp. (Fig. 3). However, this selective predation of *B. meridionalis* on chironomids was only observed at the subfamily and genus levels but not at the family level (*D* = −0.03 ± 0.11; *P *= 0.77; Fig. 3).

Fish density had no effect on diet diversity or on mean prey selectivity (Mann–Whitney tests, *P* > 0.8). Macroinvertebrate composition of gut contents did not differ between fish density treatments (Adonis, *F *= 0.94, *P *= 0.48).

### Trait‐based diet analysis

**Figure 4:**
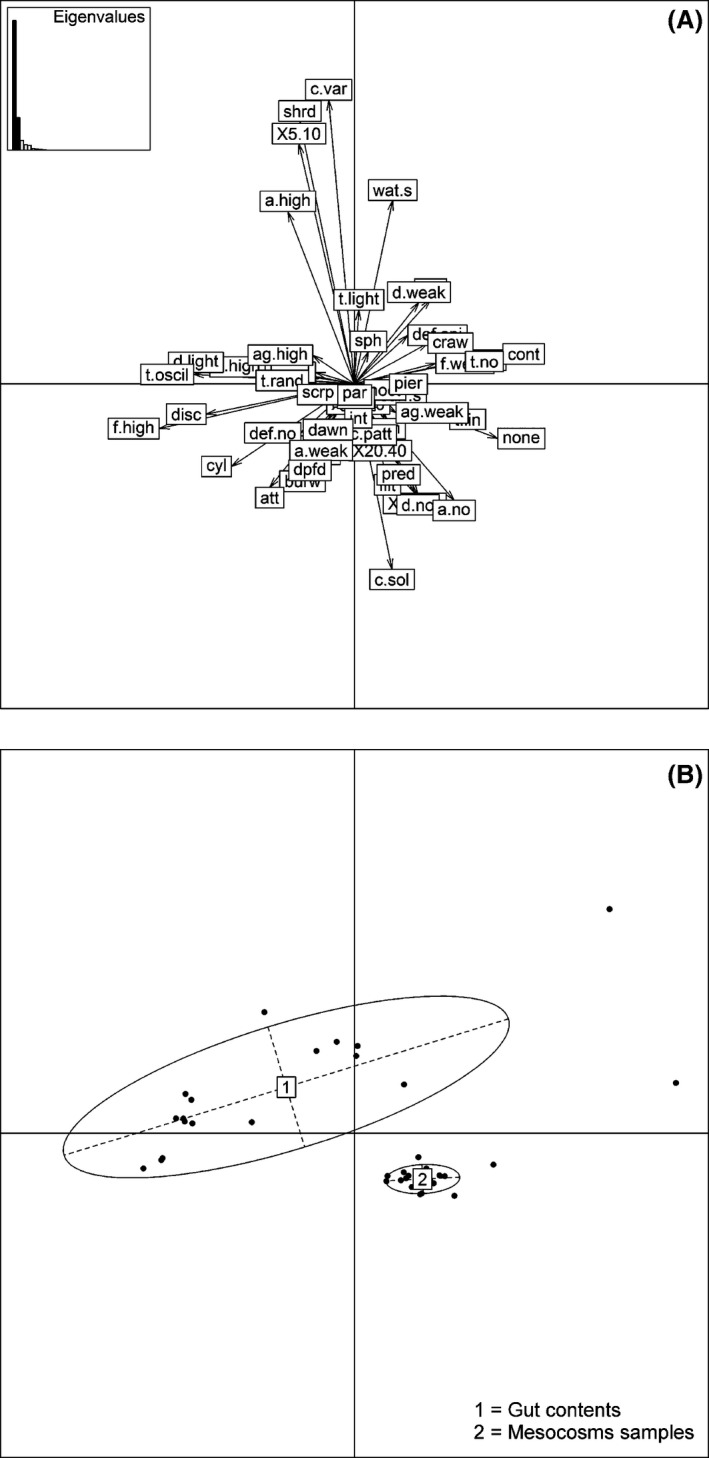
FPCA of the mesocosm samples and gut contents. (A) Plot of the distribution of the trait categories according to the mesocosm and diet samples (for codes see Table 1) and histogram of the eigenvalues. (B) Plot of the distribution of the samples; ellipses envelop weighted average of samples positions; labels “1” for gut contents and “2” for mesocosm samples indicate the gravity center of the ellipses.

In the FPCA plots of the combined datasets for mesocosm samples and gut contents (Fig. 4), the first axis with an eigenvalue of 0.046 explained a major part (69%) of the total inertia (0.066), whereas the second axis contained less information (17%). The plots showed a clear separation between mesocosm and diet samples regarding trait composition. Trait categories of “concealment ability,” “feeding habits,” “potential size,” “agility,” and “locomotion” were most prominent, but many categories seemed to have some explanatory power.

**Figure 5:**
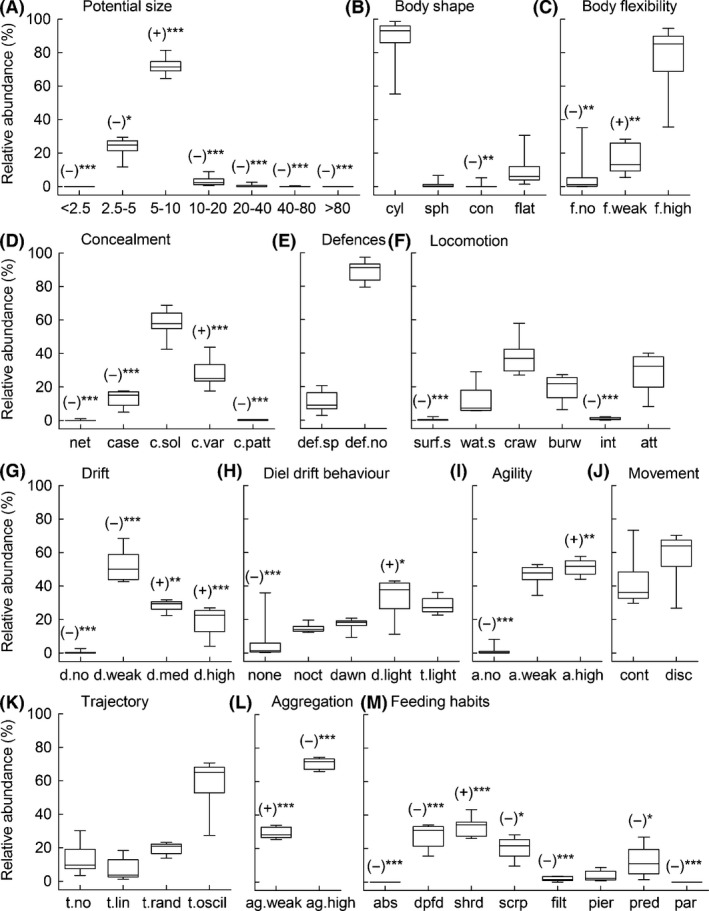
Relative importance of the 55 categories within the 13 studied traits in the *Barbus meridionalis* gut contents (for codes, see Table 1). (A) Potential size. (B) Body shape. (C) Body flexibility. (D) Cocealment. (E) Morpohological defenses. (F) Locomotion and substrate relation. (G) Tendency to drift in the water column. (H) Diel drift behaviour. (I) Agility. (J) Movement frequency. (K) Trajectory on the bottom substratum o in the drift. (L) Aggregation tendency. (M) Feeding habits. Vertical boxes represent the median, 10th, 25th, 75th, and 90th percentiles with error bars. Significant selectivity of trait categories is marked as follows: (+) positive, (−) negative or no response; the statistically significant thresholds are the following: *P < 0.05, **P < 0.01, ***P < 0.001.

The selective predation of *B. meridionalis* for particular taxa was mirrored in the trait approach. *Barbus meridionalis* significantly selected 10 of the 13 traits tested (Fig. 5). Specifically, fish fed mostly on macroinvertebrates with a potential size between 5 and 10 mm (72.5 ± 1.3%), with a cylindrical body shape (87.8 ± 3.2%), and high body flexibility (77.6 ± 4.3%). In contrast, smaller and bigger potential prey, prey with conical shape (*D* = −0.83 ± 0.12, *P* < 0.001), and prey without body flexibility (*D* = −0.47 ± 0.14, *P* = 0.006) were less vulnerable to fish predation. Macroinvertebrate concealment ability also had a significant effect on barbel selectivity. Fish fed mostly on solidly colored prey (58.2 ± 1.8%) and selected positively variable colored prey (*D* = 0.53 ± 0.04, *P* < 0.001), whereas prey with patterned color or with fixed or movable accessories (i.e., nets, retreats, cases, tubes) were less vulnerable to fish predation (Fig. 5). Most prey did not have morphological defenses (88.8 ± 1.6%), but *B. meridionalis* did not significantly select this trait category.

Regarding macroinvertebrate locomotion, crawlers were the most abundant in gut contents (38.0 ± 2.4%) followed by attached macroinvertebrates, burrowers, and full water swimmers. However, fish negatively selected surface swimmers (*D* = −0.78 ± 0.10, *P* < 0.001) and interstitial macroinvertebrates (*D* = −0.56 ± 0.06, *P* < 0.001). Interestingly, prey with high aggregation tendency dominated gut contents (70.7 ± 0.8%), but fish positively selected prey with weak aggregation tendency (*D* = 0.13 ± 0.02, *P* < 0.001). Similarly, barbels positively selected prey with medium (*D* = 0.09 ± 0.02, *P* = 0.003) and high drift tendency (*D* = 0.40 ± 0.05, *P* < 0.001), even though those with weak drift tendency predominated in fish guts (52.1 ± 2.2%). In particular, macroinvertebrates that drift during daylight were positively selected (*D* = 0.16 ± 0.06, *P* = 0.030). Most prey eaten by *B. meridionalis* also had a discontinuous movement (58.1 ± 3.3%) with an oscillatory trajectory (59.0 ± 3.2%), but these traits (movement frequency and trajectory) were not retained as significant. In contrast, prey was selected by *B. meridionalis* in relation to their swimming or crawling speed, highly agile macroinvertebrates being positively selected (*D* = 0.15 ± 0.03, *P* < 0.001) and more often captured (51.1 ± 1.5%) than slow‐moving prey which were less vulnerable (*D* = −0.80 ± 0.09, *P* < 0.001). According to trophic guilds, shredders were the most abundant feeding group in gut contents (33.2 ± 1.3%) followed by deposit feeders (27.6 ± 1.7%), scrapers (20.91 ± 1.85%), and predators (12.7 ± 2.2%). However, *B. meridionalis* only had selectivity for shredders (*D* = 0.63 ± 0.02, *P* < 0.001).

**Figure 6:**
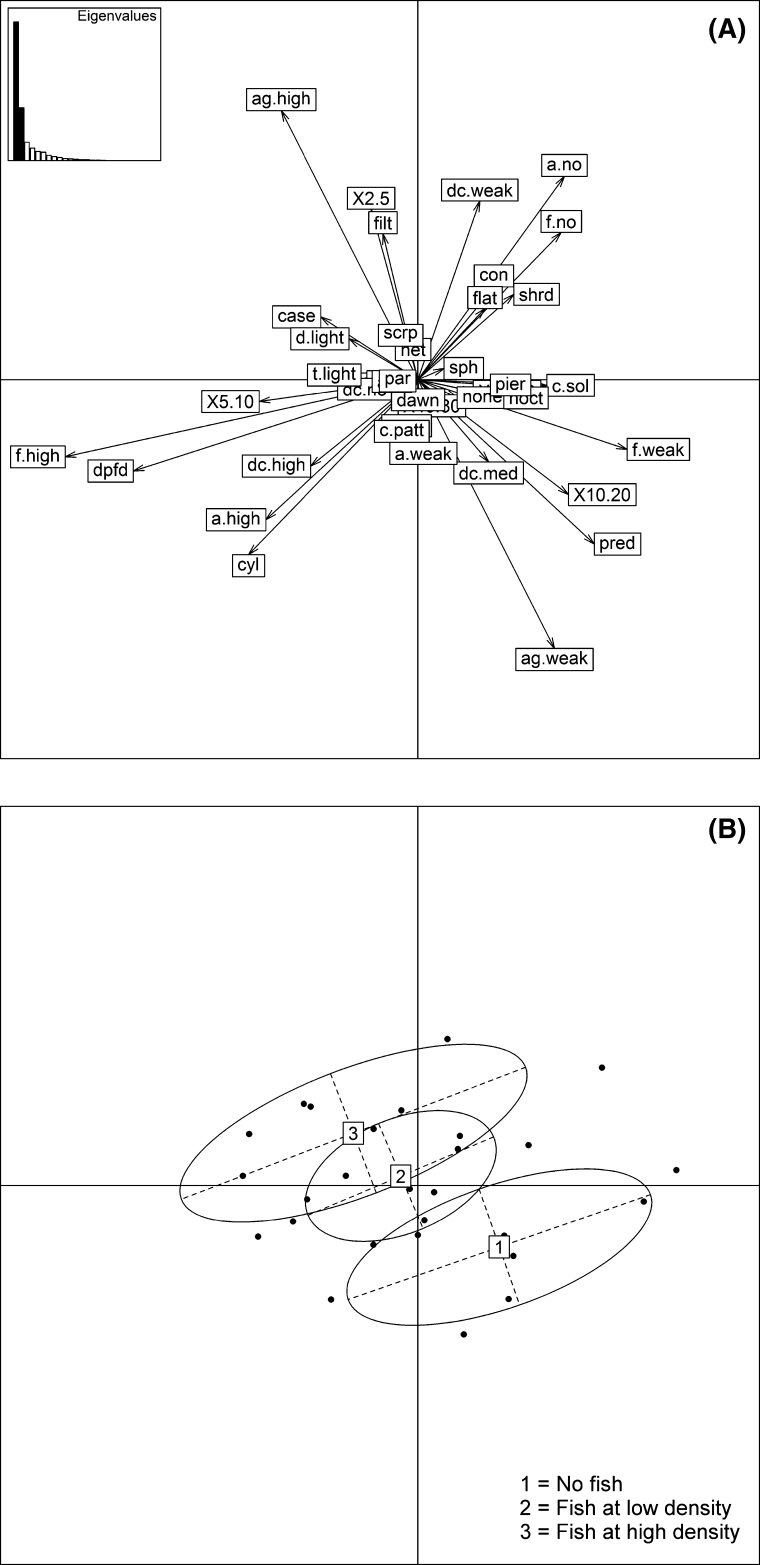
FPCA of the mesocosm samples. (A) Plot of the distribution of the trait categories according to the mesocosm samples (for codes see Table 1) and histogram of the eigenvalues. (B) Plot of the distribution of the samples; ellipses envelop weighted average of samples positions; labels “1” for the samples from the mesocosms without fish, “2” for the samples from the mesocosms with fish at low density, and “3” for the samples of the mesocosms with fish at high density indicate the gravity center of the ellipses.

In the FPCA plots of the mesocosm samples (Fig. 6), the first axis explained the 66% of the total inertia (0.002), whereas the second axis explained the 22%. The plots showed a clear effect of fish density on trait composition of the macroinvertebrate communities. Trait categories of “aggregation tendency,” “potential size,” “feeding habits,” and “body flexibility” were most prominent and related with the changes in the macroinvertebrate communities across the fish density treatments.

## Discussion

Our study shows that food choice of *B. meridionalis* can be explained by prey morphological and behavioral traits. Several studies have used trait approaches to describe fish diet (e.g., de Crespin de Billy and Usseglio‐Polatera 2002; Sánchez‐Hernández et al. 2012) and to measure food choice based on prey size (e.g., Rincón and Lobón‐Cerviá 1999; Sánchez‐Hernández and Cobo 2015). However, only some recent study has examined predator selectivity on several prey traits (Green and Côté 2014; Worischka et al. 2015). Here, we used the Jacob's D electivity index, widely used in food choice studies (e.g., Copp et al. 2005; Winkelmann et al. 2007; Lee and Suen 2014), as a simple tool to examine predator selectivity on prey traits. The benefits of examining predator selectivity with a trait approach are evident in our study. For instance, the fact that more than 88% of ingested macroinvertebrates had no morphological defenses (i.e., cerci, silk, spines) could be interpreted as fish avoidance of prey with morphological defenses. However, our analysis did not show a negative selection for this trait, suggesting that prey vulnerability to predation by *B. meridionalis* was not related to the presence of these morphological defenses.

### Taxon‐based diet analysis

Our results were consistent with previous studies showing that *B. meridionalis* mostly feed on the larvae of chironomids (*Cricotopus* spp. and *Zavrelimyia* sp.) and mayflies (*Habrophlebia* sp.) followed by coleoptera larvae (*Stictonectes* sp. and *Agabus* sp.) and hemipterans (*Parasigara* sp.) (García‐Berthou 1994; Mas‐Martí et al. 2010). Similarly, other invertebrates besides insects were also found in the guts examined in our study (e.g., gastropods), supporting that *B. meridionalis* is invertivorous (Doadrio et al. 2011). The presence of detritus and sand in the gut contents is likely to be attributed to the fact that fish ingested fine sediment unintentionally while feeding on benthic invertebrates.

In the current study, the selectivity of *B. meridionalis* for macroinvertebrate taxa was both positive and negative but mean prey selectivity was predominantly negative, as previously reported (Mas‐Martí et al. 2010). Our results show that several morphological and behavioral prey traits may explain this. Further explanations may include low energetic value and/or low palatability of some taxa (Sánchez‐Hernández et al. 2011). Last but not least, the taxonomic resolution can influence our ability to infer fish feeding preferences, such as shown here with the Chironomidae family example. *B. meridionalis* selectivity for chironomids at subfamily and genus levels was obscured when the family level was used. As chironomid taxa differ in microhabitat use, fish predation is likely to vary accordingly. For example, *Cricotopus* spp. usually live on top of stones and are, therefore, more exposed to *B. meridionalis* predation than other chironomids found underneath the stones such as *Tanytarsus* sp. and *Dicrotendipes* sp. Therefore, taxonomic resolution may be a key factor in food choice studies, as reported for food web metrics (Thompson and Townsend 2000).

*Barbus meridionalis* diet did not differ between the density treatments, contrarily to our hypotheses. Previous studies have showed that individual diet breath may increase because of inclusion of suboptimal prey when preferred prey become scarce, for example, with high intraspecific competition (Werner and Hall 1974; Martinussen et al. 2011). Therefore, our results suggested low intraspecific competition even when using high fish densities for this intermittent stream. Nevertheless, higher competition is likely to occur if *B. meridionalis* density reaches up to 20 individuals m^−2^; a condition that may take place during drought in isolated pools.

### Trait prey selectivity

We provide new insights into prey–consumer interactions suggesting that up to 10 prey traits of 13 tested influenced food choice. Our results are consistent with the OFT (Pyke 1984) as prey without body flexibility are difficult to handle which explains its low vulnerability to predation. Similarly, prey with nets, cases, or colored patterns seemed to reduce *B. meridionalis* predation by increasing prey concealment. Prey with a potential size of 5–10 mm were highly selected by *B. meridionalis*, whereas those both larger and smaller were avoided. Larger prey were most likely negatively selected because fish were gape‐limited. These results highlight the importance of size refugia for prey (Chase 1999; Woodward et al. 2010) and suggest that body size could act as a bottleneck in diet choice.

Our results also indicated that surface swimmers and interstitial taxa escaped from fish predation when compared to crawlers, burrowers, and attached organisms, most likely due to the benthic feeding preferences of *B. meridionalis* (Doadrio et al. 2011). However, pelagic taxa (i.e., full water swimmers) also occurred in the guts examined in this study, and were not negatively selected by *B. meridionalis*, indicating that *B. meridionalis* is benthopelagic. Interestingly, contrasting patterns were found regarding *B. meridionalis* selectivity for macroinvertebrates according to their aggregation behavior. While taxa with high aggregation tendency predominated in the guts examined (>70%), the electivity index showed that *B. meridionalis* preferred those with weak aggregation tendency. Although aggregate assemblages make the group more conspicuous to predators, aggregation may be an antipredator adaptation to dilute the predation impact among neighbors (Wrona and Dixon 1991) and to respond faster to detecting danger (Johannesen et al. 2014). Regarding prey feeding habits, shredders were the most vulnerable to predation, suggesting that fish preferred to forage on leaf litter substrates. As shredders have an outstanding role in the litter decomposition process, our results suggest that the loss of top‐down control by *B. meridionalis* may favor shredder activity and hence accelerate litter decomposition (Konishi et al. 2001; Boyero et al. 2008).

The presence of *Barbus meridionalis* may trigger a trophic cascade changing macroinvertebrate community composition and ecosystem function (Rodríguez‐Lozano et al. 2015). Our results showed how the feeding selectivity may also alter the functional structure of the macroinvertebrate communities. In the presence of fish, the proportion of small prey with high aggregation tendency may increase, whereas population declines and local extinctions of this fish species may favor the taxa with weak aggregation tendency, weak flexibility, and a relatively large size (10–20 mm of potential size). Besides, predatory invertebrates may increase in the absence of fish (i.e., mesopredator release).

## Conclusions

Although body size was already reported as an important determinant factor for food choice, our study suggests that up to 10 traits may be involved in the feeding preferences of *B. meridionalis*. Our results also depicted some discrepancies with the existing literature in the habitat use and diet of this fish species, highlighting the need for more basic research into the biology and ecology of species. This also applies to macroinvertebrates to build more comprehensive public trait depositories (e.g., Vieira et al. 2006; Bonada and Dolédec 2011; Sánchez‐Hernández et al. 2011). Finally, our study shows that the trait‐based perspective overcomes the limitations of the taxon‐ and size‐based approaches, improving our mechanistic understanding of predator–prey interactions and helping predict the ecological outcomes of predator invasions and extinctions.

## Data Accessibility

Data used in analyses traits of macroinvertebrates uploaded as online supporting information (Table S1, Supporting Information). Gut contents data uploaded as online supporting information (Table S2, Supporting Information) and mesocosm macroinvertebrate community data can be found in the Supporting Information of the open access publication: Rodríguez‐Lozano et al. (2015).

## Conflict of Interest

None declared.

## Supporting information


**Table S1.** Traits of macroinvertebrates.

**Table S2.** Diet of *Barbus meridionalis* in Vall d'Horta stream: abundance (%) and frequency of occurrence (%) of the main food components in fish gut contents. The different prey items are ordered by frequency of occurrence.
