# Supplementary material for: A trait‐based approach reveals the feeding selectivity of a small endangered Mediterranean fish
Source: Ecol Evol. 2016 Apr 12;6(10):3299–310. doi: 10.1002/ece3.2117 (PMC4870214; doi:10.1002/ece3.2117)
Supplement: Supplementary file 2 — Table S2. Diet of Barbus meridionalis in Vall d'Horta stream: abundance (%) and frequency of occurrence (%) of the main food components in fish gut contents. The different prey items are ordered by frequency of occurrence. [file ECE3-6-3299-s002.docx]

**Supporting Information**

Table S2. Diet of *B. meridionalis* in Vall d’Horta stream: abundance (%) and frequency of occurrence (%) of the main food components in fish gut contents. The different prey items are ordered by frequency of occurrence.

| Taxa | Abundance (%) | Frequency of occurrence (%) |
| --- | --- | --- |
| *Cricotopus* spp. | 51.1 | 100.0 |
| *Habrophlebia* sp. | 15.1 | 100.0 |
| *Zavrelimyia* sp. | 10.9 | 94.4 |
| *Stictonectes* sp. | 3.1 | 88.9 |
| *Corynoneura* sp. | 3.6 | 83.3 |
| *Microtendipes* sp. | 2.8 | 83.3 |
| *Parasigara* sp. | 3.5 | 61.1 |
| *Agabus* sp. | 1.0 | 61.1 |
| *Procladius* sp. | 0.9 | 44.4 |
| *Dicrotendipes* sp. | 1.2 | 38.9 |
| *Tanytarsus* sp. | 0.3 | 33.3 |
| *Chalcolestes viridis* | 0.3 | 33.3 |
| *Gyraulus* sp. | 1.7 | 27.8 |
| *Cladocera* | 0.4 | 27.8 |
| *Nebrioporus* sp. | 0.4 | 22.2 |
| *Baetis* sp. | 0.3 | 22.2 |
| *Gyrinus* sp. | 0.2 | 22.2 |
| *Tinodes* sp. | 0.3 | 16.7 |
| *Cloeon* sp. | 0.2 | 16.7 |
| *Sympetrum* sp. | 0.2 | 16.7 |
| Terrestrial | 0.2 | 11.1 |
| *Aeshna* sp. | 0.2 | 11.1 |
| *Nanocladius* sp. | 0.1 | 11.1 |
| *Paratanytarsus* sp. | 0.1 | 11.1 |
| Coenagrionidae | < 0.1 | 11.1 |
| *Yola bicarinata* | < 0.1 | 11.1 |
| *Physella* sp. | 0.7 | 5.6 |
| *Radix* sp. | 0.2 | 5.6 |
| Ostracoda | 0.2 | 5.6 |
| *Stictochironomus* sp. | 0.1 | 5.6 |
| *Haliplus* sp. | 0.1 | 5.6 |
| *Polypedilum* sp. | < 0.1 | 5.6 |
| *Phaenopsectra* sp. | < 0.1 | 5.6 |
| *Brillia* sp. | < 0.1 | 5.6 |
| *Helobdella stagnalis* | < 0.1 | 5.6 |
| *Rheotanytarsus* sp. | < 0.1 | 5.6 |
| *Parametriocnemus* sp. | < 0.1 | 5.6 |
| *Oulimnius* sp. | < 0.1 | 5.6 |
